# Insulator defect detection in severe weather using improved YOLOv8

**DOI:** 10.1371/journal.pone.0333175

**Published:** 2025-09-25

**Authors:** Jia Li, Yanjie Wu, Shaojun Zhu

**Affiliations:** School of Information and Control Engineering, Jilin University of Chemical Technology, Jilin, China; China Construction Fourth Engineering Division Corp. Ltd., CHINA

## Abstract

Insulators, as a vital component of the power system, encounter issues such as misdetection, leakage, and low detection accuracy in inclement weather. To address this problem, this paper proposes a YOLOv8-based insulator defect detection algorithm, YOLOv8-SSF. Firstly, SimAM (parameter-free attention mechanism) is included in the algorithm’s backbone network, which improves the ability to focus on critical features while maintaining a lightweight model. Secondly, the SPDConv layer is added to enhance the algorithm’s feature extraction capability for small-size defective targets. Furthermore, the Focal_EIOU loss function, which balances high- and low-quality anchors to increase detection and localization accuracy, replaces the CIOU loss function. According to experimental results, the enhanced algorithm reduces the rate of misdetection and omission of defects on transmission conductors, accomplishes a comprehensive simultaneous improvement, and achieves 87.2% mean average accuracy (mAP@0.5) on the dataset.

## Introduction

The robust operation of the power system is a key factor in ensuring the stable development of human life and production. Insulators are indispensable components in the power system [1], which not only support high-voltage transmission lines but also ensure the safe isolation of the current from the ground or other conductors. However, insulators subjected to severe conditions over an extended duration may incur a range of failures, including flashover and breakage [2]. It is imperative that power systems are capable of rapid and accurate detection in order to identify insulators and their defects. This is of particular importance as it is directly related to the safe and stable operation of the power grid [3]. The conventional approach to insulator inspection is predicated on human inspection, a method that is frequently inefficient and vulnerable to human factors. This inherent susceptibility engenders challenges in ensuring the accuracy and consistency of the inspection outcomes [4].

In the domain of insulator defect recognition, researchers have proposed a range of innovative methods and techniques to address the challenges of achieving optimal detection accuracy and efficiency. These methodologies can be categorized into two distinct approaches: two-stage algorithms (two-stage) and single-stage algorithms (one-stage) [5]. The two-stage algorithms first extract the object region and then classify the region for recognition. The traditional algorithms are Fast-RCNN [6] and Faster-RCNN [7], along with other families. While such algorithms have been shown to be highly accurate, they are inherently slow due to the requirement of a two-stage computation process. In the context of insulator defect detection, it is imperative to address latent hazards in a timely manner to ensure the reliable functioning of the power system. Consequently, the necessity for expedited detection procedures is paramount [8]. The single-stage algorithm has been developed for the purpose of generating candidate target frames directly on the input image. These frames are then classified and recognized. Representative algorithms for this purpose include the YOLO series [9]. This algorithm demonstrates a high level of detection speed; however, its recognition accuracy is not optimal in cases of image blurring and when dealing with small targets. It is imperative that power system inspection be an accurate identification of insulator defects; only then can the purpose of inspection be achieved. Consequently, further optimization of this type of algorithm is required [10].

Therefore, this paper proposes an improved YOLOv8-SSF insulator defect detection algorithm, which focuses on small target defects in harsh environments, reduces their leakage rate, and improves the accuracy of insulator defect detection. The improvements made in this paper are as follows:

- In addressing the challenges associated with insulator defect detection in harsh environments, which are characterized by a high propensity for misdetection, omission, and the degradation of image quality, a space-to-depth SPDConv convolution module is incorporated into the backbone network. This integration serves to rectify the loss of fine-grained information and the paucity of learning features, thereby enhancing the detection capability of insulator defects of minimal size and insulator images of reduced resolution.
- The SimAM parameter-free attention mechanism is introduced to achieve the synergistic work of channel attention and spatial attention, thereby effectively suppressing the background features and improving the feature extraction capability of the model for insulator defects without adding additional parameters.
- In addressing the imbalance issue in the regression of the original YOLOv8 model, the loss function of the original model is substituted for the loss function of Focal_EIOU. This enhances the focus on high-quality anchors in the regression process, reduces the discrepancy between the width and height of the actual box and the predicted box, and reduces the impact of anomalous boxes on the identification of insulators.

## Related work

The field of deep learning has seen significant advancement and improvement in recent years, and more researchers are using this cutting-edge technology to tackle target detection problems. With the rise of deep learning, there has been a marked progression in this advanced technology to address challenges. This phenomenon has garnered significant attention within academic discourse.

Chen Y et al. [11] proposed a lightweight Faster-RCNN (Faster-RCNNtiny) defect detection model for insulators. The model replaces ResNet with EfficientNet in the backbone network and employs feature pyramids to construct feature fusion feature maps with different resolutions. This approach significantly reduces the model parameters while improving the detection accuracy. However, the study lacks an analysis of the confusion matrix. Yu Z et al. [12] proposed an insulator defect recognition algorithm based on the YOLOv5 model. This pioneering algorithm proposes an adaptive neighborhood-weighted median filtering (NW-AMF) noise reduction algorithm, improves the new lightweight network structure RcpVGG-YOLOv5, and introduces the loss function EIOU. The latter optimizes the penalty for the edge length of the target frames and improves the contribution of the high-quality target gradient. The dataset is of limited size, and there is an absence of a detection strategy for small target insulation.

Liu D. [13] proposed a YOLOv8 algorithm incorporating a bottleneck-like feature pyramid (BiFPN) and an attention mechanism, as well as a lightweight decoupling head for the detection of defects in overhead insulators. This algorithm achieves a lightweight design while improving the detection accuracy. However, it should be noted that the algorithm is only able to detect a single type of insulator breakage in the dataset. Yingming Gao et al. [14] proposed an improved target detection algorithm for YOLOv8 insulators and their self-exploding defects. The algorithm adds a small target detection header to the original structure, with the aim of encouraging the model to pay more attention to small targets. It also adds deformable convolution and an inverse residual attention module in the backbone network, with the intention of solving the problem of targeting the part of the insulator pictures in the dataset that have complex backgrounds and are covered with occlusions. However, the dataset has limitations and lacks severe insulator pictures under inclement weather.

Liu Hang et al. [15] proposed a lightweight-based improved YOLOv8n transmission line insulator self-destruction detection method. The YOLOv8n network is taken as the base model, with its detection capability enhanced by the incorporation of a small target detection module. This module is designed to capture detailed information regarding insulator self-explosion. Furthermore, the SIoU loss function is introduced to address the limitation of the original CIoU loss function, which fails to consider the direction between the real frame and the predicted frame. This enhancement is intended to improve target localization accuracy. Finally, the channel pruning method is employed to reduce the model’s computational cost and complexity. Boya Yuan proposed a novel GER-YOLO algorithm for the detection of insulator defects [16]. Firstly, GhostNetV2 is employed to construct the C2fGhostV2 module, which is capable of maintaining the algorithm’s detection accuracy while significantly reducing the number of parameters and computations. Subsequently, an efficient multi-scale attention (EMA) network with cross-space learning capability is introduced with a view to fully mining feature information while suppressing redundant information. Finally, the C2fRFE module is proposed to further capture long-range information and learn multi-scale features to improve the detection capability of insulators and their defects at different scales.

Li D et al. [17] proposed a multi-scale insulator defect detection method using a Detection Transformer (DETR) to address the issue of capturing insulator defects amidst the rapid movement of unmanned aerial vehicles (UAVs) in adverse weather conditions such as rain, snow, and fog. This method aims to enhance the accuracy of defect detection. Firstly, a multi-scale backbone network is proposed to effectively capture the features of small targets. Furthermore, a self-attention up-sampling (SAU) module has been incorporated to supersede the conventional attention module, thereby augmenting the system’s capacity for contextual information extraction and small target detection. Finally, the Insulator Defect Loss Function (IDIoU) is introduced to mitigate the instability in the matching process caused by small defects. Feng et al. [18] proposed an improved network to address the issue of insulator defects being difficult to detect in UAV images due to variable scales and complex backgrounds. Firstly, an attention mechanism is introduced into the backbone network with a view to attenuating the influence of the background in the target area. Secondly, in conjunction with the bidirectional feature pyramid network structure, cross-layer connectivity and weighted fusion are performed during feature extraction and fusion, thereby enabling the network to focus more acutely on insulator defect features and to suppress the influence of noise. Finally, the detection head in the lowest layer is replaced with a small target detection head, thus enhancing the network’s focus on small targets.

For data enhancement, Yuxuan Wang et al. [19] proposed a Lifespan Automatic Data Augmentation (ALADA) framework. In this study, a lifetime search space is formulated to efficiently sample enhancement strategies and generate enhancement images for joint optimization. Secondly, the authors propose a three-step, bi-level optimization scheme as an alternative to the retraining strategy. This scheme alternately updates the model and the enhancement parameters, thereby reducing the workload of hyperparameter tuning during retraining using the search strategy. Finally, to solve the non-trivial problem of joint optimization, strategic gradient sampling is employed to efficiently estimate the gradient flow. Waqar Ahmed Khan [20] proposed the BLWELM (balanced WELM) algorithm, which combines the WELM algorithm for k-relearning with multiple sampling methods to improve decision boundaries, reduce data complexity, and obtain better class distribution results. This solves the problem of data imbalance.

Although the above improved algorithm, based on deep learning, enhances the detection accuracy of insulator defects to a certain extent, it is affected by factors such as the diversity of insulator defects. This results in the model’s accuracy for detecting insulator defects being compromised.

## Proposed methodology

### Introduction to the YOLOv8 improved model

**Fig 1:**
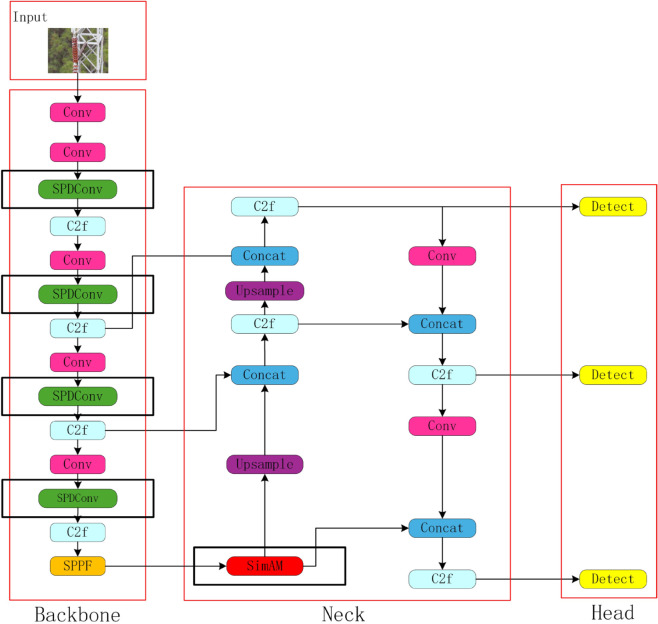
Structure diagram of the improved YOLOv8 network model.

The primary network structure of YOLOv8 comprises the Backbone, Neck and Head [21] with the principal modules illustrated in Fig 1. This configuration enhances the detection speed and accuracy in comparison with YOLO series algorithms such as YOLOv5 and YOLOv7. However, there is some leakage and misdetection for small targets of the insulator type. In order to address the challenges previously mentioned, this paper proposes an improved detection algorithm, YOLOv8-SSF. The algorithm provides tailored enhancements to the backbone network of YOLOv8 to address the problem of missing information at fine granularity and improves the feature extraction capability for small target insulators in complex backgrounds. Furthermore, the employment of the Focal_EIOU loss function as a replacement for the CIOU loss function of the original model serves to enhance the consistency between the true and predicted frames, thereby improving the model’s accuracy.

### Parameter-free attention mechanism

**Fig 2:**
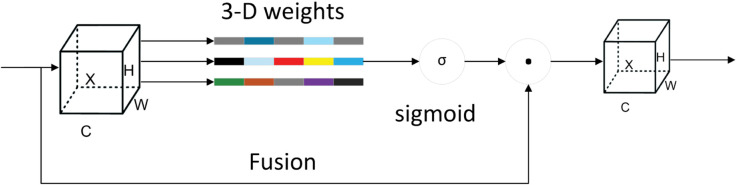
Structural diagram of the SimAM attention mechanism.

Inspired by the ability of humans to efficiently localize to critical regions in complex scenes, attention mechanisms have been introduced in the field of computer vision to enhance model performance and efficiency [22]. While traditional channel attention (1-D) and spatial attention (2-D) mechanisms have been extensively utilized, they typically necessitate a substantial number of parameters and a sequence of intricate operations, thereby augmenting the complexity of the model and often concentrating on a single form of attention [23]. In contrast, the SimAM attention mechanism adopted in this paper is a lightweight 3-D attention mechanism without additional parameters [24]. The model synthesizes the synergistic effects of channel attention and spatial attention, enhancing its coordination and efficiency in processing the task without the introduction of additional task parameters. The network structure of the SimAM attention mechanism is depicted in Fig 2. The importance of each neuron is evaluated by defining an energy function $e_{t}^{*}$, the mathematical expression of which is shown in Eqs (1)–(3):

$$e_{t}^{*}=\frac{4({\hat{\sigma }}^{2}+\lambda )}{(t-\hat{\mu }{)}^{2}+2{\hat{\sigma }}^{2}+2\lambda }$$ (1)

$$\hat{\mu }=\frac{1}{M}\sum_{i=1}^{M}X_{i}\;$$ (2)

$${\hat{\sigma }}^{2}=\frac{1}{M}\sum_{i=1}^{M}(X_{i}-\hat{\mu }{)}^{2}\;$$ (3)

In this equation, t denotes the target neuron, *μ* denotes the mean of all neurons except t, ${\hat{\sigma }}^{2}$ denotes the variance of all neurons except t, M denotes the number of neurons, and *λ* is the canonical term.

In cases where the value of $e_{t}^{*}$ is smaller, the neuron in question is more distinct from peripheral neurons and more important for visual processing. Therefore, the importance of each neuron is expressed by the reciprocal of the minimum energy, and the larger the reciprocal, the higher the importance. The final SimAM attention module expression is as follows:

$$\tilde{X}=sigmoid(\frac{1}{E})⊙X$$ (4)

X denotes the input feature map, and E signifies the set of all neuron values $e_{t}^{*}$ of the input feature map. The sigmoid function is incorporated to impose constraints on the values in E, thereby ensuring that they do not exceed certain thresholds.

As posited by SimAM, the assignment of fine-grained weights to deep semantic features is achieved by means of a dynamic adjustment of the weights of pixels in the feature map. This process serves to highlight the target region and attenuate background noise interference. As demonstrated in Fig 1, the enhanced model incorporates the SimAM attention mechanism within the connecting region of the backbone and neck, with the objective of optimizing the efficacy of information transfer. The SimAM attention mechanism further refines the captured multiscale spatial information, transforming it into more precise and discriminative features through the attention mechanism. This refinement contributes to an enhancement in the overall performance of the model.

### SPDConv

In conditions of extreme climate, the images captured by insulators frequently demonstrate a propensity to exhibit characteristics associated with low resolution. Moreover, insulator defects are typically characterized by their diminutive size, rendering them challenging to detect. However, conventional Convolutional Neural Networks (CNN), which demonstrate efficacy in the processing of images of moderate size and good resolution, exhibit a substantial decline in their detection performance when confronted with the task of image blurring or small object size [25]. In order to surmount this challenge, reference is made to the SPDConv convolution module [26] in the original network with a view to enhancing the detection of small targets and low-resolution images.

**Fig 3:**
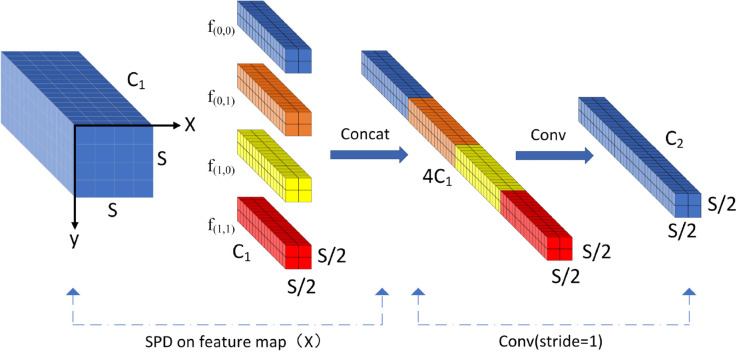
SPDConv structure diagram.

SPDConv is composed of a space-to-depth (SPD) layer and a non-strided-convolution (Non-strided-Conv) layer, which serves to reduce the computational complexity by decomposing the standard convolution operation. The latter decomposes the convolution in spatial dimensions into the convolution of multiple smaller convolution kernels. This decomposition is shown to preserve the spatial sensory field of the original convolution and to enhance the feature extraction capability of the model for small targets [26]. Its structural configuration is illustrated in Fig 3. For flash defects, the spatial decomposition operation of SPDConv is equivalent to multi-scale texture analysis of the local region. Processing the four neighbors separately enables sub-feature maps to be captured and texture gradient changes and intensity distributions in different directions to be identified. The non-spanning convolutional layer adaptively fuses this multi-angle texture information through learnable parameters. This optimizes the ability to perceive low-contrast, gradient-varying dirt patterns and overcomes the problem of insufficient texture feature extraction in blurred images using traditional convolution. For geometric defects such as breakage, SPDConv reduces the resolution while maintaining the integrity of the shape by utilizing redundancy in the channel dimension. Unlike conventional downsampling, which results in the loss of geometric information in small broken regions, SPDConv redistributes spatial information to the channel dimension, ensuring edge and contour information is retained. The non-spanning convolutional layer uses a channel combination approach to learn shape features that specifically enhance detection sensitivity to irregular edges and broken contours.

**Fig 4:**
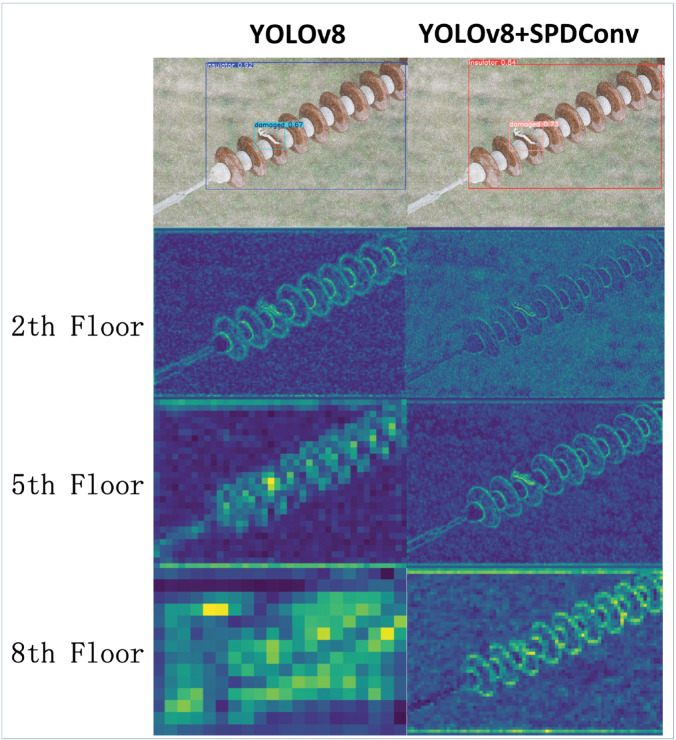
Comparison of model feature extraction before and after adding SPDConv.

To compare the impact on the model’s learning ability more intuitively before and after the SPDConv module is added, the feature maps of layers 2, 5, and 8 are visualized, as shown in Fig 4. From Fig 4, it can be seen that after the introduction of SPDConv, the detailed features of the feature maps acquired by the model are more obviously enriched.

In the case of scale=2, the original feature map (S, S, C1) is defined as such: S denotes the height and width, and C1 denotes the number of channels. The data is sliced along the x, y directions, yielding four sub-feature maps of size (S/2, S/2, C1). These are then subjected to a downsampling operation. All sub-feature maps are spliced along the channel dimension in order to obtain a feature map X′ that is two-thirds less than the original feature map in spatial dimensions and four times more than the original feature map in channel dimensions (S/2, S/2, 4C1). The output feature map X″ (S/2, S/2, C2) is then input into a non-spanning convolutional layer containing C2 filters. The fundamental formula is presented in Eq (5). The variables fx and y are comprised of all i plus x and i plus y, respectively, which can be divided by the ratio scale. The SPDConv convolution with scale=2 is introduced into the original network model with a view to enhancing the feature extraction capability for low-resolution images and small targets under inclement weather and improving the recognition rate of insulator defects.

$$\left\{ \begin{array}{c} f_{0,0}=X[0:S:2,0:S:2]\\ f_{1,0}=X[1:S:2,0:S:2]\\ f_{0,1}=X[0:S:2,1:S:2]\\ f_{1,1}=X[1:S:2,1:S:2] \end{array}\right.$$ (5)

### Focal_EIOU

The CIOU loss function [27] employed in YOLOv8 is inadequate in accurately measuring the discrepancy between the target frame and the actual frame. It is also less effective in detecting small targets with category imbalance, which complicates the problem of small-target occlusion. Furthermore, insulator defects are susceptible to being overlooked and misidentified in inclement weather conditions. The solution to this problem is presented in this paper through the introduction of the Focal_EIOU loss function [28]. This function has two primary functions: firstly, it minimizes the difference between the width and height of the real frame and the predicted frame, thus accelerating convergence; secondly, it eliminates the interference caused by anomalous frames during the identification of the insulator, thus improving the detection accuracy of small targets. The Focal_EIOU loss function formulae are shown in Eqs (6)–(8):

$$L_{EIOU}=L_{IOU}+L_{dis}+L_{asp}\;=1-IOU+\frac{\rho^{2}(b,b^{gt})}{(w^{c}{)}^{2}+(h^{c}{)}^{2}}+\frac{\rho^{2}(w,w^{gt})}{(w^{c}{)}^{2}}+\frac{\rho^{2}(h,h^{gt})}{(h^{c}{)}^{2}}$$ (6)

$$L_{Focal\_EIOU}=IOU^{\gamma }L_{EIOU}$$ (7)

$$IOU=\frac{|A\cap B|}{|A\cup B|}$$ (8)

Where $\rho^{2}(b,b^{gt})$ denotes the distance between the prediction frame and the center point of the real frame, c is the diagonal distance between the prediction frame and the smallest outer closed region of the real frame, the parameters *wgt*, *hgt*, w, and h denote the width and height of the real frame and the width and height of the prediction frame of the detected object, respectively, A and B denote the volume of two arbitrary shapes of the measurements, and *γ* is the parameter controlling the degree of suppression of the anomalies.

## Experimental analysis

### Experimental environment and parameters

In this paper, the configuration of the experimental environment is carried out on the Linux Ubuntu operating system, with PyTorch utilized as the deep learning framework. The specific experimental environment configuration is illustrated in Table 1. The resolution of the input image is 640×640 pixels, and during the training process, the number of samples selected in each round of batch_size is set to 16; the SGD optimizer is selected, and the initial learning rate is 0.001; the number of training rounds is 200.

**Table 1:** Experimental environment configuration.

Operating System	Linux Ubuntu
GPU	NVIDIA GeForce RTX 4090
GPU Memory	24G
Python Version	3.10
Pytorch Framework	2.2.2

### Datasets

**Fig 5:**
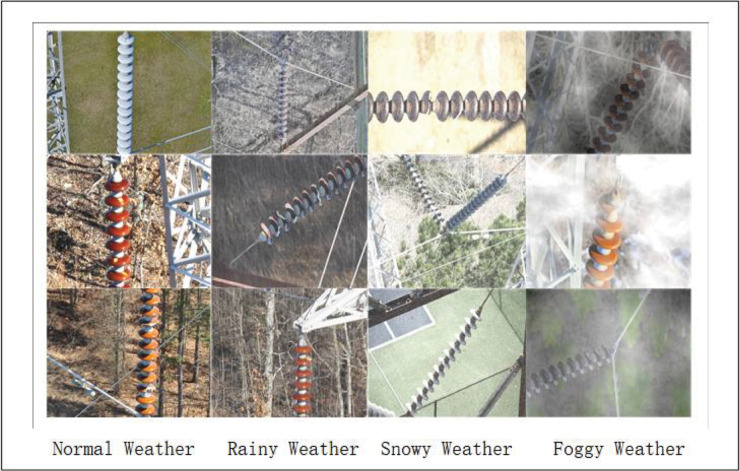
Insulator dataset data enhancement.

In this paper, the ‘The insulator defect image dataset (IDID)’, a publicly available dataset comprising 1,600 high-resolution images of insulators, damage and flashover, is employed [29]. The images are divided into three distinct classes. In the context of experimental research, the scarcity of data can impede the model’s capacity for effective generalization. This limitation arises due to the undersampling of data points, resulting in a lack of diversity in the training dataset. Consequently, the model may overfit to specific inputs, leading to suboptimal performance and inaccurate experimental results. Therefore, the dataset is expanded by means of a series of operations, including brightness enhancement, cropping, and rotation. On this basis, the dataset is expanded to include rain, snow, fog, and other inclement weather, with the aid of algorithms. The resultant dataset comprises 4,500 pictures, as illustrated in Fig 5. The dataset is then divided into three sets: the training set, the test set, and the validation set, according to a ratio of 8:1:1.

### Evaluation indicators

The paper employs Average Precision (AP), Recall (R), Precision (P), and Mean Average Precision (mAP) as the evaluation metrics for network model performance. The calculation formula is as follows:

$$Precision=\frac{TP}{TP+FP}$$ (9)

$$Recall=\frac{TP}{TP+FN}$$ (10)

$$AP=\int_{0}^{1}P\;\text{d}R$$ (11)

$$mAP=\frac{\sum_{i=1}^{N}AP}{N}$$ (12)

In this study, TP is defined as the number of correctly detected samples, FP as the number of incorrectly detected samples, FN as the number of missed samples, and AP as the area enclosed by the precision-recall (P-R) curve and the axes. The mean value of AP for all categories is denoted mAP. The mAP@0.5 metric, which denotes the average precision calculated at an IoU threshold of 0.5, and the mAP@0.5:0.95 metric, which denotes the average precision calculated at an IoU threshold ranging from 0.5 to 0.95, with the average precision calculated between 0.05 intervals, are of particular interest. The number of categories, N, is also a relevant factor.

## Experimental results

### Confusion matrix analysis

**Fig 6:**
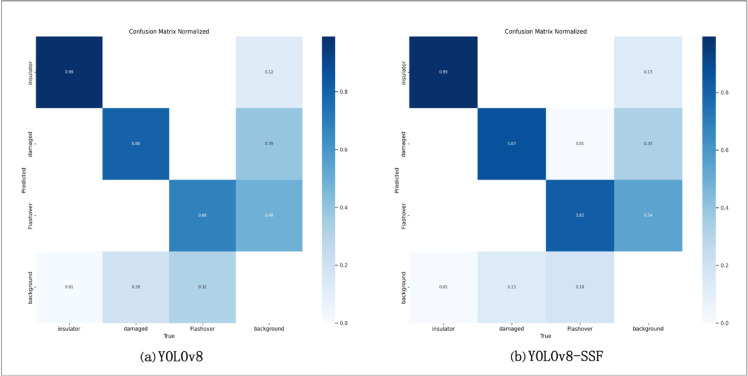
Confusion matrix diagram of the original and improved algorithms.

A confusion matrix is a visualization tool used in supervised learning to show how an algorithm’s actual predictions correspond to the classification results in a dataset. Analyzing the confusion matrix provides insight into the accuracy with which the improved algorithm recognizes each category. This allows for a more comprehensive understanding of the model’s actual performance, identifying its strengths and weaknesses and facilitating the evaluation of its overall and category-specific performance. In Fig 6, Fig (a) shows the results of YOLOv8 on the insulator dataset. As seen in Fig (a), the accuracy is 99% for normal insulators, 80% for insulator breakage, and 68% for insulator flashover. Fig (b) shows the confusion matrix results for YOLOv8-SSF, which shows 99% accuracy for normal insulators, 87% accuracy for insulator breakage, and 82% accuracy for insulator flashover. In terms of normal insulator accuracy, both models perform well, but the improved YOLOv8-SSF algorithm achieves a 7% improvement in insulator breakage and a 14% improvement in insulator flashover compared to YOLOv8. The enhanced YOLOv8-SSF algorithm’s superior performance was verified by comparing the confusion matrices of the insulator dataset before and after the improvement.

### Comparative experiments

**Fig 7:**
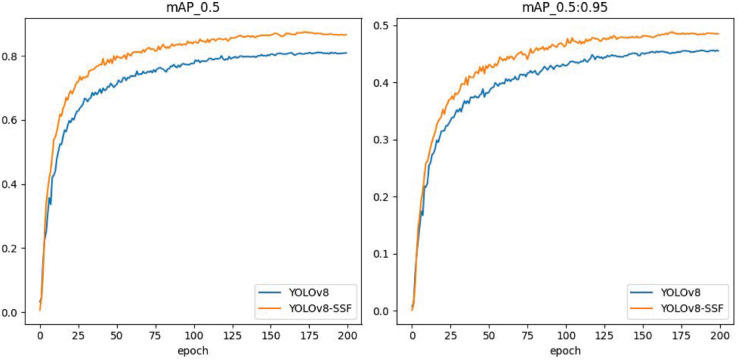
Comparison of mAP@0.5 and mAP@0.5:0.95 curves.

To provide a more comprehensive evaluation of the effectiveness of the paper’s algorithm, YOLOv8-SSF, for insulator defect detection, the algorithm is compared with some mainstream algorithms in a set of comparison experiments. In these experiments, the Faster-RCNN [7] from the two-stage algorithms and the YOLOv8 [21], YOLOv5 [30], YOLOv10 [31] and YOLOv11 [32] from the one-stage algorithms, as well as the Transformer-based RT-DETR [33] target detector, were chosen. The findings are presented in Table 2, which illustrates that the mAP@0.5 of the enhanced YOLOv8-SSF algorithm has been elevated by 5.4%, 2.3%, 2.2%, 5.1%, 7.7% and 4.2% in comparison with the conventional algorithms YOLOv8, YOLOv5, YOLOv10, and Faster-RCNN. This substantial enhancement serves as a testament to the efficacy of the proposed methodology in this study. The two-stage algorithm Faster-RCNN exhibits the lowest mAP@0.5, a consequence of the network structure of the model that results in a substantial loss of information. In addition, the YOLOv8-SSF algorithm obtained the maximum value of 48.8% for mAP@0.5:0.95, in comparison to the values achieved by other networks. The performance advantages of this paper’s algorithm in the insulator defect detection task are further verified through comparison experiments of a series of mainstream algorithms mentioned above. The mAP@0.5 and mAP@0.5:0.95 curves of the original model and the improved model are shown in Fig 7. It is evident from this figure that, as the number of model training rounds increases, the mAP value of YOLOv8 also increases. The mAP@0.5 reaches 0.818, and the mAP@0.5:0.95 reaches 0.446. It is clear that the mAP value of the improved model is much higher than that of the original model. Ultimately, mAP@0.5 can attain 0.872, while mAP@0.5:0.95 can achieve 0.488.

**Table 2:** Results of the comparative experiment.

Algorithmic	AP_0.5_(%)			mAP@0.5(%)	mAP@0.5:0.95(%)
	Insulator	Damaged	Flashover		
Faster-RCNN	95.6	78.5	62.3	79.5	43.5
RT-DETR	97.4	83.8	68.6	83.0	45.4
YOLOv8	97.8	79.5	65.8	81.8	44.6
YOLOv5	98.6	82.8	73.3	84.9	46.4
YOLOv10	99.2	83.8	72.1	85.0	48.4
YOLOv11	99.2	79.3	67.8	82.1	46.4
Ours	99.1	84.6	77.9	87.2	48.8

While the paper compares well with models based on the YOLO architecture, it lacks comparisons with other deep learning algorithms. Therefore, the Swin Transformer model (referred to as Swin-T in the table) is chosen for comparison with the model in the paper [34], and the results are shown in the Table 3 below.

**Table 3:** Model comparison experiments.

Algorithmic	AP_0.5_(%)			mAP@0.5(%)
	Insulator	Damaged	Flashover	
Swin-T	98.9	84.9	70.6	84.8
Ours	99.1	84.6	77.9	87.2

As can be seen from the table, although the AP value of the ‘Damaged’ class of the Swin Transformer model is higher than that of this paper’s algorithm, the AP values of the other two classes are lower. Furthermore, the AP value of the ‘Flashover’ class is 7.3% lower. Furthermore, the mAP value of the Swin Transformer model is only 0.848, which is 2.4% lower than that of this paper. These results prove that this paper’s algorithm is superior to the Swin Transformer model.

### Ablation experiments

In order to verify the feasibility of the improved YOLOv8-SSF algorithm, an experimental approach was adopted involving the implementation of an ablation experiment. This entailed the integration of the enhanced module on the foundation of the original YOLOv8 model. The efficacy of each enhanced module was then analyzed in relation to the algorithm. The evaluation results are displayed in Table 3, and ✓ represents the incorporation of this module. As demonstrated in Table 4, the incorporation of the loss function Focal_EIOU leads to a 3% enhancement in mAP@0.5 compared to the baseline model. Additionally, P and R exhibit respective improvements of 3.6% and 3.5%, signifying that the loss function minimizes the disparity between the width and height of the real and predicted frames, thereby enhancing the accuracy of detecting small targets. The mAP@0.5 demonstrated a 3.1% enhancement following the incorporation of the SimAM attention mechanism into the model, thereby exhibiting enhanced feature extraction capabilities for the detection of small targets. The incorporation of SPDConv in Backbone has been shown to enhance mAP@0.5, P, and R by 4.5%, 3.4%, and 5.3%, respectively. This finding lends further credence to the hypothesis that SPDConv convolution is a more efficacious approach for the detection of image blurring and the analysis of small object size. As evidenced by the final two rows of the table, the combination of improvement points has been shown to enhance the fine-tuning accuracy of the models. The YOLOv8-SSF model has been demonstrated to achieve the highest values for both mAP@0.5 and P and R.

**Table 4:** Results of ablation experiments.

Model	Focal_EIOU	SimAM	SPDConv	mAP@0.5(%)	P(%)	R(%)
YOLOv8				81.8	86.5	89.0
YOLOv8	✓			82.1	90.1	92.5
YOLOv8		✓		82.2	87.7	92.8
YOLOv8			✓	86.3	90.6	94.3
YOLOv8	✓		✓	86.7	89.9	94.6
YOLOv8	✓	✓	✓	87.2	91.2	95.0

### Loss function comparison experiment

**Fig 8:**
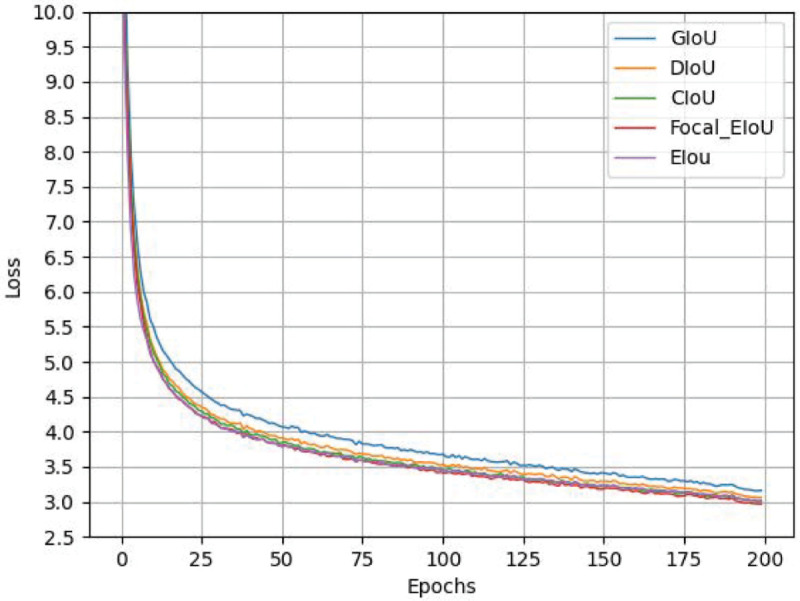
Loss curve comparison diagram.

The CIOU loss function [27] utilized in the original YOLOv8 model is inadequate in its sensitivity to small targets when targeting small target tasks, and it is unable to effectively measure the difference between the target frame and the real frame. Consequently, the Focal_EIOU loss function is introduced. As illustrated in Table 5, the localization errors of the disparate loss functions (GIOU [35], DIOU [27], and EIOU [28]) on the target are presented, alongside the mAP@0.5 of Focal_EIOU, which is observed to be the highest in comparison with the other loss functions. Furthermore, the Box_loss bounding box loss function is the lowest, with a reduction from 84.8% to 82.5% compared with the original model and an improvement in localization accuracy of 2.3%. Consequently, the Focal_EIOU loss function, which has been substituted, can locate insulator defects with greater precision. The loss function graph is illustrated in Fig 8.

**Table 5:** Comparison results of the loss functions.

Loss Functions	mAP@0.5(%)	b_box
GIOU	80.5	85.3
DIOU	80.4	85.1
CIOU	81.8	84.8
EIOU	78.7	84.1
Focal_EIOU	82.1	82.6

### Comparative experiments on attention mechanisms

In this paper, we propose the incorporation of the SimAM participantless attention mechanism within the YOLOv8 model. To ascertain the impact of varying attention mechanisms on the model, we have implemented several common attention mechanisms, namely SA [36], CBAM [37], and CA [38], within the same position of SimAM in the YOLOv8 backbone. The experimental results are displayed in Table 6. In comparison with SimAM, the incorporation of three additional attention mechanisms results not only in an augmentation of the model’s parameters but also in a reduction in accuracy. Specifically, the accuracy of the SA attention mechanism is 4.2% lower than that of the SimAM attention mechanism, and SimAM itself is 0.4% higher than the original YOLOv8 model, without any increase in the number of parameters. The SimAM parameter-less attention mechanism facilitates the collaborative operation of the spatial attention mechanism and the channel attention mechanism, thereby enhancing the detection accuracy of insulator defects without any increase in the number of parameters.

**Table 6:** Comparison of attention mechanisms.

Attention mechanisms	Parameters	mAP@0.5(%)
YOLOv8	3011433	81.8
SA	3033405	78.0
CBAM	3072123	78.1
CA	3024913	80.4
SimAM	3011433	82.2

To validate the sensitivity analysis based on changing the position of the SimAM attention mechanism within the model in the same environment and to prove that the current position is optimal, the following experiments are conducted in this paper. The results are shown in the Table 7 below:

**Table 7:** Comparison of attention mechanisms.

Attention mechanisms	Parameters	mAP@0.5(%)	mAP@0.5:0.95(%)
9th Floor	3011433	81.2	45.8
13th Floor	3033405	80.9	45.0
17th Floor	3072123	81.0	45.4
Ours	3011433	82.2	46.1

As can be seen from the table, the model size of this attention mechanism remains unaffected when it is added at different positions. However, the values for mAP@0.5 and mAP@0.5:0.95 when SimAM attention is added to the 9th, 13th, and 17th layers are lower than the values in this paper for the position where the attention is added. The mAP@0.5 for the 13th layer is 0.809, which is 1.3% lower than in this paper, and the mAP@0.5:0.95 is 0.450, which is 1.1% lower than in this paper. The sensitivity analysis of the above experiments proved that the current added position is optimal.

### Analysis of detection effect

**Fig 9:**
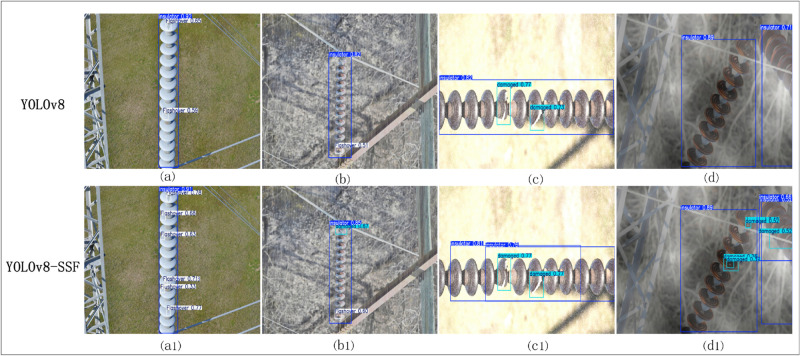
Visualisation and analysis of test results.

In order to visually compare the detection effect of the original YOLO model with the improved YOLOv8-SSF model, the insulator defect images processed in section “Section 4.2” are used for testing and performance comparison under the same hardware environment. The results are displayed in Fig 9. In the insulator dataset, (a), (b), (c), and (d) denote YOLOv8 detections in normal, rain, snow, and fog environments, and (a1), (b1), (c1), and (d1) denote YOLOv8-SSF detections in these environments. As demonstrated in Fig.8, the original YOLOv8 model exhibits leakage detection issues in both normal environments and inclement weather. In contrast, the enhanced YOLOv8 model demonstrates the capability to detect all insulator defect targets. The enhanced YOLOv8 model exhibits superior detection accuracy for insulator defects in comparison to the original YOLOv8 model. This finding serves to substantiate the enhanced algorithm’s proficiency in identifying insulator defects within intricate scenarios, thereby underscoring its remarkable anti-interference capacity.

## Conclusion

This paper presents the findings of a study conducted on insulator defect recognition in the context of transmission lines. The study revealed that the current methods employed in this field are characterized by low accuracy, with issues including misdetection and omission in the detection process. This paper proposes an algorithm for detecting insulator defects. The algorithm is based on an improved version of the YOLOv8 model, to which a SimAM parameter-free attention mechanism is added to the backbone network. This is intended to enhance the model’s ability to extract features while ensuring that the model remains lightweight. The Focal_EIOU loss function is used as the regression task loss to reduce the difference between the real and predicted frame widths and heights. This is intended to improve the model’s detection accuracy. Finally, the SPDConv is introduced to enhance the model’s ability to detect small targets and low-resolution insulator images. The findings indicate that the enhanced YOLOv8-SSF algorithm is more effective in addressing the aforementioned issues. Specifically, mAP@0.5, P, and R demonstrate enhancements of 4.5%, 3.4%, and 5.3%, respectively, in comparison to the original YOLOv8. In contrast to alternative prevalent models, the algorithm exhibits a substantial enhancement in detection accuracy. Consequently, the enhanced YOLOv8 network model is particularly well-suited for the detection of insulator defects.

## References

[1] Teng L, Tian H. Damage detection of insulators in catenary based on deep learning and Zernike moment algorithms. Applied Sciences. 2022;12(10):5004. doi: 10.3390/app12105004

[2] Liu C, Yi W, Liu M. A lightweight network based on improved YOLOv5s for insulator defect detection. Electronics. 2023;12(20).

[3] Wang H, Wang K. Real-time detection method of insulator defects under haze based on dmsanet-yolov7. Radio Engineering. 2024;54(06):1431–9.

[4] Li Z, Li B. Detection of anti-vibration hammer defects in transmission lines based on multi-scale convolutional attention mechanism. Journal of Electrotechnology. 2024;39(11):3522–37.

[5] Békési GB. Benchmarking generations of you only look once architectures for detection of defective and normal long rod insulators. J Control Autom Electr Syst. 2023;34(5):1093–107. doi: 10.1007/s40313-023-01023-3

[6] Girshick BR, Donahue J, Darrell T. Rich feature hierarchies for accurate object detection and semantic segmentation. CoRR. 2013.

[7] Ren S, He K, Girshick R, Sun J. Faster R-CNN: towards real-time object detection with region proposal networks. IEEE Trans Pattern Anal Mach Intell. 2017;39(6):1137–49. doi: 10.1109/TPAMI.2016.2577031 27295650

[8] Yang Z, Liu Y, Li X. Deep learning based insulator fault detection method for transmission lines. Journal of Electrical Engineering. 2024;19(02):325–34.

[9] Redmon J, Divvala KS, Girshick BR. You only look once: unified, real-time object detection. CoRR. 2015. doi: abs/1506.02640

[10] Wen B, Hu Y, Peng S. A lightweight network for multi-defect detection of transmission line insulators. Radio Engineering. 2024:1–9.

[11] Chen Y, Deng C, Sun Q. Lightweight detection methods for insulator self-explosion defects. Sensors. 2024;24(1):290-. 10.3390/s24010290 PMC10781199 38203151

[12] Yu Z, Lei Y, Shen F. Research on identification and detection of transmission line insulator defects based on a lightweight YOLOv5 network. Remote Sensing. 2023;15(18).

[13] Liu D. Study on insulator defect detection based on improved YOLOv8. Journal of Physics: Conference Series. 2024;2770(1).

[14] GAO Y, HAN S, ZHOU B. Improved YOLOv8 algorithm for insulators and their self-explosion detection. Radio Engineering. 2024:1–10.

[15] Liu H, Li M, Liu Z. Improved YOLOv8n insulator self-destruct defect detection method based on. Journal of Electronic Measurement and Instrumentation. 2025;39(01):57–69. doi: 10.13382/j.jemi.B2407642

[16] Yuan B, Li Y, Ye Q. GER-YOLO defect detection algorithm for transmission lines. Advances in Lasers and Optoelectronics. 2024;61(22):149–59.

[17] Li D, Yang P, Zou Y. Optimizing insulator defect detection with improved DETR models. Mathematics. 2024;12(10):1507.

[18] Feng F, Yang X, Yang R. An insulator defect detection network combining bidirectional feature pyramid network and attention mechanism in unmanned aerial vehicle images. Engineering Applications of Artificial Intelligence. 2025;152:110745.

[19] Wang Y, Chung SH, Khan WA. ALADA: A lite automatic data augmentation framework for industrial defect detection. Advanced Engineering Informatics. 2023;58:102205.

[20] Khan WA. Balanced weighted extreme learning machine for imbalance learning of credit default risk and manufacturing productivity. Annals of Operations Research. 2025;348(2):833–61.

[21] Sun H, Tan C, Pang S. RA-YOLOv8: an improved YOLOv8 seal text detection method. Electronics. 2024;13(15):3001.

[22] Guo M-H, Xu T-X, Liu J-J, Liu Z-N, Jiang P-T, Mu T-J, et al. Attention mechanisms in computer vision: a survey. Comp Visual Med. 2022;8(3):331–68. doi: 10.1007/s41095-022-0271-y

[23] YAO J, CHENG G, WAN F. Improved YOLOv8 defect detection algorithm for lightweight bearings. Computer Engineering and Application. 2024:1–13.

[24] Yang L, Zhang RY, Li L. Simam: a simple, parameter-free attention module for convolutional neural networks. In: International Conference on Machine Learning. 2021. p. 11863–74.

[25] Cui Y, Li J, Hou L. Improved YOLOv7 target detection method for urban small UAVs. Journal of Computer Engineering & Applications. 2024;60(10).

[26] Zhichao H, Yi W, Junping W. Improved lightweight rebar detection network based on YOLOv8s algorithm. Advances in Computer, Signals and Systems. 2023;7(10).

[27] Zheng Z, Wang P, Liu W. Distance-IoU loss: faster and better learning for bounding box regression. Proceedings of the AAAI Conference on Artificial Intelligence. 2020;34(07):12993–3000.

[28] Yi-Fan Z, Weiqiang R, Zhang Z. Focal and efficient IOU loss for accurate bounding box regression. Neurocomputing. 2022. p. 146–57.

[29] Lewis D, Kulkarni P. Insulator defect detection. IEEE Dataport. 2021.

[30] Yang W, Liu T, Zhou J. A CNN-Swin Transformer forest wildlife image target detection algorithm based on improved YOLOv5s. Forestry Science. 2024;60(03):121–30.

[31] Wang A, Chen H, Liu L. Yolov10: real-time end-to-end object detection. arXiv preprint. 2024. doi: arXiv:2405.14458

[32] Khanam R, Hussain M. Yolov11: an overview of the key architectural enhancements. arxiv preprint. 2024. doi: arxiv:2410.17725

[33] Zhao Y, Lv W, Xu S. Detrs beat yolos on real-time object detection. In: Proceedings of the IEEE/CVF Conference on Computer Vision and Pattern Recognition. 2024. p. 16965–74.

[34] Liu Z, Lin Y, Cao Y. Swin transformer: hierarchical vision transformer using shifted windows. In: Proceedings of the IEEE/CVF International Conference on Computer Vision. 2021. p. 10012–22.

[35] Rezatofighi HS, Tsoi N, Gwak J. Generalized Intersection over Union: A Metric and A Loss for Bounding Box Regression. CoRR. 2019. doi: abs/1902.09630

[36] Zhang QL, Yang YB. Sa-net: Shuffle attention for deep convolutional neural networks. In: ICASSP 2021 -2021 IEEE International Conference on Acoustics, Speech and Signal Processing (ICASSP). 2021. p. 2235–9.

[37] Woo S, Park J, Lee JY. Cbam: convolutional block attention module. In: Proceedings of the European Conference on Computer Vision (ECCV). 2018. p. 3–19.

[38] Hou Q, Zhou D, Feng J. Coordinate attention for efficient mobile network design. In: Proceedings of the IEEE/CVF Conference on Computer Vision and Pattern Recognition. 2021. p. 13713–22.

